# Positive allosteric modulation of P2X7 promotes apoptotic cell death over lytic cell death responses in macrophages

**DOI:** 10.1038/s41419-019-2110-3

**Published:** 2019-11-25

**Authors:** Stefan Bidula, Kshitija Dhuna, Ray Helliwell, Leanne Stokes

**Affiliations:** 1 School of Pharmacy, University of East Anglia, Norwich Research Park, Norwich, United Kingdom; 2 School of Health and Biomedical Sciences, RMIT University, Bundoora, VIC 3083 Australia

**Keywords:** Apoptosis, Cell death and immune response

## Abstract

P2X7 is an ATP-gated ion channel that is highly expressed by leukocytes, such as macrophages. Here, P2X7 has been demonstrated to be involved in the regulation of various cell death pathways; including apoptosis, pyroptosis, necrosis, and autophagy. However, cell death induction via P2X7 is complex and is reliant upon the nature of the stimulus, the duration of the stimulus, and the cell type investigated. Previous reports state that high extracellular ATP concentrations promote osmotic lysis, but whether positive allosteric modulation of P2X7 in the presence of lower concentrations of ATP condemns cells to the same fate is unknown. In this study, we compared cell death induced by high ATP concentrations, to cell death induced by compound K, a recently identified and potent positive allosteric modulator of P2X7. Based on our observations, we propose that high ATP concentrations induce early cell swelling, loss of mitochondrial membrane potential, plasma membrane rupture, and LDH release. Conversely, positive allosteric modulation of P2X7 primarily promotes an intrinsic apoptosis pathway. This was characterised by an increase in mitochondrial Ca^2+^, accelerated production of mitochondrial ROS, loss of mitochondrial membrane permeability in a Bax-dependent manner, the potential involvement of caspase-1, and caspase-3, and significantly accelerated kinetics of caspase-3 activation. This study highlights the ability of positive allosteric modulators to calibrate P2X7-dependent cell death pathways and may have important implications in modulating the antimicrobial immune response and in the resolution of inflammation.

## Introduction

P2X7 is an ATP-gated ion channel expressed predominantly on immune cells such as macrophages. Here, it plays key roles within inflammation via the activation of the NLRP3-caspase-1 inflammasome, release of pro- and anti-inflammatory mediators (e.g. IL-1β, IL-18, Annexin A1), shedding of transmembrane proteins (i.e. CD62L, CD23), and regulation of cell death processes (necrosis, apoptosis, pyroptosis, autophagy)^1^. To date, the involvement of P2X7 in regulating cell death has proven to be complex and varied, and is governed by the cell type, nature of the stimulus, and duration of the stimulus^2^. Prolonged activation of P2X7 has been linked to typical morphological changes during apoptosis, such as cell blebbing, cell shrinkage, nuclear fragmentation, and chromatin condensation^3^. Moreover, P2X7 can regulate cell death via caspase-8 and caspase-9; stimulating ROS production, mitochondrial dysfunction, cytochrome c release, and caspase-3/7 activation^3–6^. Conversely, P2X7 can also activate the canonical (caspase-1 dependent) pyroptosis pathway in LPS-primed cells. Here, ATP can function as a damage-associated molecular pattern (DAMP), resulting in cell blebbing, cytoplasmic swelling, release of cytokines and alarmins, and ultimately lytic cell death^7–10^. Furthermore, ATP can activate the non-canonical pyroptosis pathway following LPS priming. In this pathway caspase-11 in mice, or caspase-4 and -5 in humans, can partially cleave pannexin-1 channels, resulting in K^+^ efflux and release of ATP into the extracellular space^11^.

P2X7 is therefore an attractive target to manipulate cell death pathways in scenarios where the type of death is important; for example, during cancer, or following infection with intracellular pathogens such as *Mycobacterium tuberculosis* or *Toxoplasma gondii*^12^. Moreover, cancer cells and pathogens can subvert the immune response by employing the use of ectonucleotidases such as CD39 and CD73 to limit the availability of ATP, or by pathogens hiding within cells to evade recognition by host immune receptors^13,14^. Therefore, the ability to enhance or accelerate P2X7-dependent cell death pathways in these scenarios could prove critical in the resolution of infection or inflammatory situations.

One way in which the activity of P2X7 can be modified is via allosteric modulation. We recently identified a novel positive allosteric pocket within the central vestibule of human P2X7 that is potentiated by ‘steroid-like’ triterpenoid glycosides, termed ginsenosides^15,16^. Particularly, compound K (CK), an in vivo metabolite from the medicinal herb *Panax ginseng* had a profound effect on P2X7-dependent responses and could potentiate ATP-induced channel opening at nanomolar concentrations^15^. Moreover, CK could enhance Ca^2+^ signalling, formation of the macropore, and enhance cell death of macrophages to a non-lethal concentration of ATP^15^. However, the mechanism of cell death regulated by P2X7 in the presence of positive allosteric modulators is currently unknown.

Our aim in this study was to compare the effects of high ATP concentrations or positive allosteric modulation by CK on the induction of P2X7-dependent cell death pathways and elucidate the cell death mechanism employed in murine macrophages. We describe a mechanism whereby positive allosteric modulation of P2X7 primarily promotes an intrinsic apoptosis pathway, characterised by an increase in mitochondrial Ca^2+^, accelerated production of mitochondrial ROS, loss of mitochondrial membrane permeability in a Bax-dependent manner, the potential involvement of caspases-1, and -3, and significantly accelerated caspase-3/7 activation.

## Materials and methods

### Cell culture

Mouse macrophage cell line J774.2 (obtained from ECACC General Cell Culture Collection, UK) were maintained in RPMI-1640 media containing L-glutamine (Life Technologies, Fisher Scientific, UK) supplemented with 10% foetal bovine serum (Sigma US origin, F2442) and 100 U/ml penicillin plus 100 µg/mL streptomycin (Fisher Scientific, UK). Cells were maintained at 37 °C in a humidified incubator supplied with 5% CO_2_. Cells were not tested for mycoplasma contamination.

For cell stimulations, stock ATP (A7699, Sigma–Aldrich, UK) was prepared as a solution of 100 mM in distilled water and pH was corrected to 7.4 with 5 M NaOH. Aliquots were frozen at −20 °C and used once. Ginsenoside CK (CAS#39262-14-1, purity >98%) was from Chemfaces, China and was prepared as 10 mM stock in DMSO.

### Flow cytometry

To quantify cell surface expression of murine P2X7 (mP2X7), 5 × 10^5^ cells were pelleted prior to resuspension in primary mouse anti-mouse P2X7 antibody (Hano43; Enzo Life Sciences, UK) at a dilution of 1:20 in cold PBS/0.5% BSA buffer. Cells were stained for 1 h on ice and then washed with PBS/0.5% BSA buffer. This was followed by staining with a goat anti-rat IgG Alexa488 secondary antibody (Fisher Scientific, UK) at 1:100 dilution for 1 h on ice. Following washing with PBS/0.5% BSA buffer, cells were re-suspended in PBS/0.5% BSA buffer for acquisition on a CytoFLEX flow cytometer (Beckman Coulter, USA; laser excitation, 488 nm; emission detection, 533/30 nm). Data were analysed using CytExpert software (Beckman Coulter; version 2.1).

### Dye uptake experiments

For YOPRO-1 dye uptake experiments cells were plated at a density of 2 × 10^4^ cells/well in complete RPMI 1640 media (100 µL per well) in poly-D-lysine coated 96-well plates. Media was removed using a manual multichannel pipette and replaced with a low divalent cation buffer (145 mM NaCl, 2 mM KCl, 13 mM D-glucose, 10 mM HEPES and 0.1 mM CaCl_2_, pH 7.3) containing 2 µM YO-PRO-1 iodide (Life Technologies catalogue number Y3663). For most experiments, ginsenosides (10 µM) were co-injected simultaneously with the agonist using a Flexstation 3 microplate reader (Molecular Devices, UK). Ginsenosides and agonist were prepared at 10X final concentration in the compound plate. Dye uptake over time was recorded using an excitation wavelength of 488 nm and an emission wavelength of 520 nm on the Flexstation 3 (6 reads/well, PMT setting medium). Basal fluorescence measurements were acquired for 40 sec followed by automatic injection of agonist and the kinetic measurement of fluorescence intensity was performed for 300 sec using Softmax Pro v5.4 software (Molecular Devices). Measurements were performed in triplicate and repeated in three independent experiments. Dye uptake responses were calculated as area under the curve from 50–300 sec using zero baseline normalised data.

### Intracellular calcium measurements

For calcium measurements cells were plated at a density of 2 × 10^4^ cells/well in complete RPMI 1640 media (100 µL per well) in poly-D-lysine coated 96-well plates. Cells were loaded with 2 µM Fura-2AM (Fisher Scientific) in HBSS buffer containing 250 µM sulfinpyrazone (Sigma–Aldrich, UK) for 40–60 min at 37 °C. Following loading, buffer was removed using a multichannel pipette and replaced with standard extracellular buffer or low divalent buffer. Cells were warmed for 10 min before measurements were started. Fura-2 was measured at excitation wavelengths 340 nm and 380 nm with emission wavelength 520 nm using a Flexstation 3 plate reader. Sampling interval was 3.5 sec and 3 reads/well. Basal fluorescence measurements were acquired for 40 sec followed by automatic injection of agonist and the kinetic measurement of fluorescence intensity was performed for 300 sec. Fura-2 ratio was calculated using Softmax Pro v5.4.5 and responses measured using area under curve kinetic reduction.

For simultaneous Fluo-4 and Rhod-2 calcium measurements, J774 cells were plated at a density of 5 × 10^4^ cells/well in complete RPMI 1640 media (100 µL per well) on poly-D-lysine coated 96-well plates. Cells were loaded with 2 µM Rhod-2AM in HBSS buffer containing 2 mM probenecid (Fisher Scientific, UK) for 60 min at 37 °C. Loading buffer was removed and replaced with HBSS containing 2 µM Fluo-4AM and 2 mM probenecid. This was incubated for 40 min at 37 °C. Loading buffer was removed and replaced with extracellular buffer containing 2 mM CaCl_2_ (zero magnesium) and 1 mM probenecid. Cells were warmed to 37 °C for 10 min in the Flexstation 3 before measurements were started. Fluo-4 fluorescence was measured at excitation 490 nm and emission 520 nm (cut-off 515 nm) using 6 reads/well on PMT medium; Rhod-2 fluorescence was measured at excitation 550 nm and emission 580 nm (cut-off 570 nm) using 6 reads/well on PMT medium using a dual wavelength protocol. Agonists were prepared at 10X final concentration and automatically injected at 30 sec. Recordings were made for 300 sec in Flex mode allowing analysis of the calcium signals immediately after injection of agonists +/− modulators. Following completion of recordings, further measurements were made in kinetic mode every 60 sec for 90 min to follow calcium changes over prolonged time. Data were collected from three independent experiments from triplicate wells. Data were analysed by quantifying the fluorescence change over 300 sec by area under curve calculations in Softmax Pro software v5.4.5 (normalised by zero baseline). Kinetic profiles follow absolute fluorescence values over the time-course of the experiment.

### Membrane potential assays

J774 cells were plated at a density of 2 × 10^4^ cells/well in complete RPMI 1640 media (100 µL per well) on poly-D-lysine coated 96-well plates. Cells were left to adhere overnight. Membrane potential blue (Molecular Devices) was prepared in standard extracellular buffer. Media was removed from the plate using a multichannel pipette and 180 µL of membrane potential blue solution added to the cells. The plate was then incubated at 37 °C in a humidified incubator for 30 min. Agonists were prepared at 10X final concentration and added to a drug plate. Fluorescence was measured using a Flexstation 3 multimode plate reader using excitation wavelength 525 nm and emission wavelength 565 nm (auto cut-off 550 nm). PMT setting was medium and 3 reads/well. Each column was read for 180 sec (2 sec intervals) and then recordings continued at a sample rate of 1 reading/60 sec for 90 min. Data were collected from three independent experiments from triplicate wells. Data were then analysed by quantifying the fluorescence change over 180 sec using area under curve calculations in Softmax Pro software v5.4.5 (normalised by zero baseline).

### Lactate dehydrogenase (LDH) assay

LDH release was quantified using a Pierce™ LDH Cytotoxicity Assay Kit (Fisher Scientific) and was conducted as per manufacturer’s instructions. In brief, 50 µL of cell supernatant was incubated with 50 µL of the LDH reaction mixture for 30 min at RT prior to measuring absorbance at 490 nm on a Flexstation 3 plate reader. To calculate toxicity, the background absorbance (spontaneous LDH release) was subtracted from the sample values. The total amount of LDH contained in the cells was obtained following lysis of the cells. The LDH absorbance values obtained from the samples were then converted and represented as a percentage of the overall LDH contained within the cells.

### Cell viability

Viability experiments were performed using the CellTiter 96^®^ Aqueous Non-Radioactive Cell Proliferation assay (Promega, UK), a colorimetric method for determining the number of viable cells. J774 cells were plated at 2 × 10^4^ cells/well in RPMI 1640 media supplemented with 1% or 10% serum and left for 24 h under normal growth conditions. Treatments were then added to the cells (2× final concentration) and incubated for a further 6 or 24 h. MTS solution (20 µL) was added to media in each well 4 h before the stipulated end point time. Absorbance was measured at 490 nm using a Clariostar plate reader (BMG Labtech, UK) or a Flexstation 3 plate reader.

We also used an AlamarBlue metabolic assay to measure cell viability. For these experiments resazurin (0.1 mg/mL in PBS; Sigma–Aldrich) was added to the cells and incubated for a further 2 h at 37 °C. Fluorescence was measured on a Flexstation 3 plate reader (excitation, 570 nm; emission, 585 nm). The background fluorescence of the media alone in the absence of cells was subtracted from all samples. Data were collected from triplicate wells for each experiment and experiments performed three independent times.

The Multi-tox-fluor multiplex cytotoxicity assay (Promega, UK) was used, which measures the activity of two proteases in both live and dead cells. The multi-tox fluor multiplex cytotoxicity assay was conducted as per manufacturer’s instructions. Equal parts of the viable cell protease substrate (GF-AFC Substrate) and the dead-cell protease substrate (bis-AAF-R110 Substrate) were mixed together in the assay buffer and 100 µL was added to cells (200 µL [300 µL total volume]) for the final 30 min of incubation time at 37 °C. Fluorescence was then measured in a Flexstation 3 (Live-cell fluorescence measured at an excitation of 400 nm and emission of 505 nm; dead-cell fluorescence measured at an excitation of 485 nm and emission of 520 nm).

To inhibit cell death, caspase inhibitors for pan-caspases, caspase-1, caspase-3, and caspase-8 (Z-VAD-FMK, (Ac-YVAD-CMK, Z-DEVD-FMK [and Ac-DEVD-CHO], and Z-IETD-FMK, respectively) or MCC950 were used at 10 µM and pre-incubated for 2 h prior to stimulation. Inhibitors were from Santa Cruz (Z-IETD-FMK and Ac-YVAD-CMK) or Bio-Techne (Z-VAD-FMK, Z-DEVD-FMK, MCC950). To scavenge mitochondrial ROS, mitoTEMPO (Sigma) was used at 25 µM, to inhibit Bax the Bax V5 peptide (Bio-Techne, UK) was used at 200 µM, to chelate Ca^2+^ EGTA was used at a concentration of 5 mM.

### Caspase-3/7 activation

Caspase-3/7 activation in cells was quantified using an EarlyTox™ Caspase-3/7 NucView 488 kit (Molecular Devices, UK) according to manufacturer’s instructions. This method utilises a substrate consisting of a fluorogenic DNA dye coupled to the caspase-3/7 DEVD recognition sequence. When the substrate permeates the plasma membrane, caspase-3/7 can cleave the substrate, releasing a high affinity dye that stains the nucleus. In brief, J774 cells were plated at 2 × 10^4^ cells/well in RPMI 1640 media supplemented with 10% serum or HBSS (Fisher Scientific, UK) supplemented with 20 mM HEPES and left for 24 h under normal growth conditions. Treatments were added to the cells (2× final concentration) and incubated for either 24 h under normal growth conditions or placed directly in an ImageXpress® Widefield system (Molecular Devices, Australia) for 12 h to measure the kinetics of caspase-3/7 activation. The caspase-3/7 NucView substrate was added to the cells at a final concentration of 5 µM, incubated at room temperature for 30 min, and protected from light. To inhibit caspase activation, cells were incubated overnight with 10 µM of the pan-caspase inhibitor Z-VAD-FMK (Bio-Techne, UK). Caspase-3/7-positive cells were observed via microscopy (FITC filter; Leica, Germany) and caspase-3/7 activation kinetics were quantified using the ImageXpress® (laser excitation, 500 nm; emission detection, 530 nm). The area of cells was quantified using Fiji software v.2.

### Measurement of reactive oxygen species (ROS)

The production of either cellular ROS or mitochondrial ROS (mtROS) was quantified using 2’,7’ -dichlorofluorescin diacetate (DCFDA; Molecular Probes, UK) or MitoSOX™ Red reagent (Molecular Probes, Fisher Scientific, UK), respectively. J774 cells were plated at 2 × 10^4^ cells/well (96-well black clear bottom plates) in phenol red-free RPMI 1640 media supplemented with 10% serum and left for 24 h under normal growth conditions. For the quantification of cellular ROS, cells were loaded with a final concentration of 5 µM DCFDA for 45 min at 37 °C prior to removal of the dye and the addition of treatments in low divalent assay buffer. DCFDA fluorescence was detected on a Flexstation 3 plate reader (excitation, 492 nm; emission, 520 nm). For the quantification of mtROS, cells were loaded with a final concentration of 5 µM MitoSOX™ Red reagent for 10 min at 37 °C prior to removal of the dye and the addition of treatments in low divalent assay buffer. MitoSOX™ red fluorescence was detected on a Flexstation 3 plate reader (excitation, 510 nm; emission, 580 nm). Readings were taken every 15 min for 60 or 105 min for cellular and mitochondrial ROS, respectively. Samples were read using PMT low and either 30 or 50 reads per well for cellular ROS and mitochondrial ROS, respectively. For the MitoSOX™ assays in the presence of 5 mM EGTA, readings were taken using kinetic mode on the Flexstation 3 with readings being taken every 60 sec for 120 min.

### Measurement of mitochondrial membrane potential

Mitochondrial membrane potential was quantified using tetramethylrhodamine methyl ester (TMRM; Molecular Probes, Fisher Scientific). J774 cells were plated at 2 × 10^4^ cells/well (96-well plate) in RPMI 1640 media supplemented with 10% serum and left for 24 h under normal growth conditions. Cells were stimulated with their respective treatments for 2, 4, or 24 h in RPMI 1640 media containing 10% serum. The media was replaced with RPMI 1640 containing 100 nM TMRM for the final 30 min of cell treatment and incubated at 37 °C. Cells were washed with pre-warmed phenol red-free RPMI and cells were visualised via fluorescent microscopy (RFP/TRITC filter, emission detection at 574 nm). The percentage of TMRM positive cells was quantified using Fiji v.2. One image per well was obtained (triplicate wells per treatment) on a Leica DM16000 inverted microscope (Hoescht exposure 888.9 ms; TMRM exposure 1.2 s) using Leica application suite version 2.8.1.

### Statistical analysis

Graphs were plotted using GraphPad Prism version 7 (La Jolla, USA). Concentration-response curves were fitted using a log (agonist) vs response–variable slope (four parameter) best-fit equation. Error bars are standard deviation. Data were analysed for statistical significance using one-way ANOVA or two-way ANOVA with post-tests as appropriate, including multiple comparisons testing (GraphPad Prism). Significance was taken as *P* < 0.05.

## Results

### CK potentiates calcium mobilisation, YOPRO-1 uptake responses, and augments cell death of J774 macrophages in a P2X7-dependent manner

**Fig. 1:**
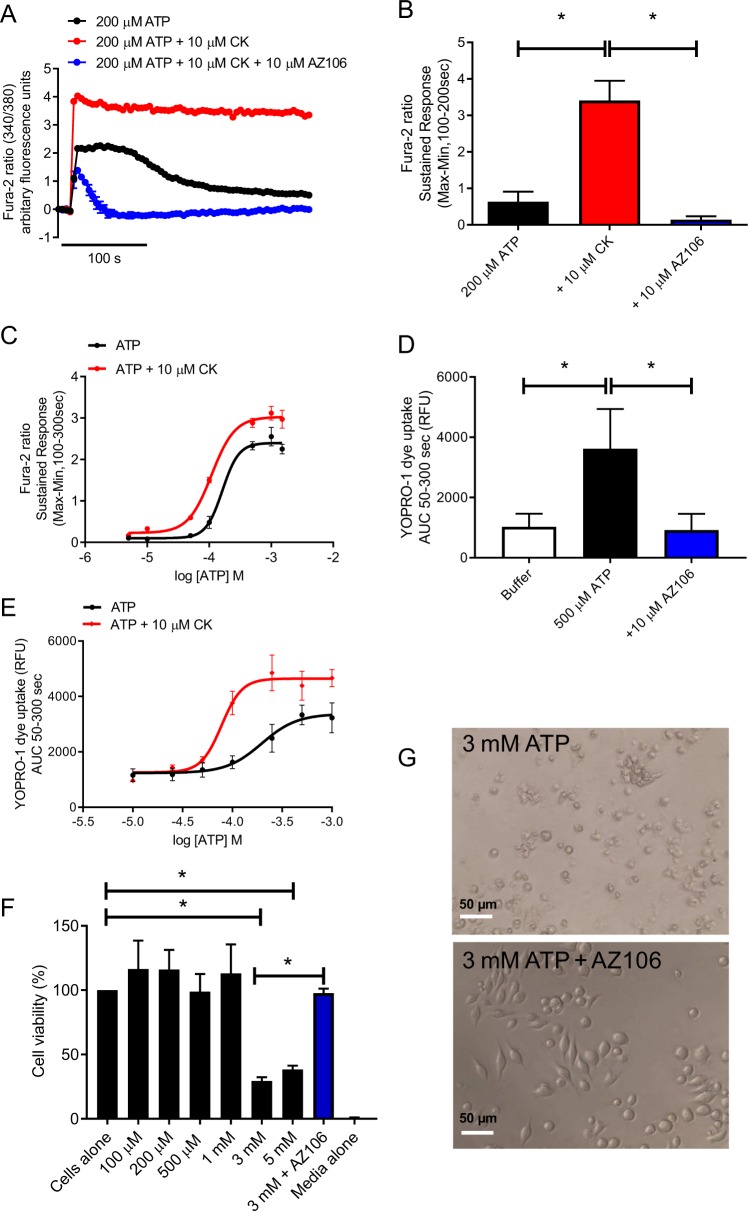
Compound K (CK) potentiates P2X7 responses. **a** J774 macrophages were stimulated with 200 µM ATP in the presence or absence of CK or CK + AZ10606120 (both 10 µM) and Ca^2+^-responses were quantified using Fura-2. **b** The sustained response (100-200 sec) was quantified as an indicator of P2X7-dependent responses. **c** J774 macrophages were stimulated with a range of ATP concentrations in the presence or absence of CK (10 µM) prior to quantification of the Fura-2 sustained response as in **b**. **c** J774 macrophages were stimulated with 500 µM ATP in the presence or absence of AZ10606120 (10 µM) to quantify uptake of YOPRO-1 dye. **d** YOPRO-1 dye uptake by J774 macrophages to a range of ATP concentrations in the presence or absence of CK (10 µM). **f** Cell viability of J774 macrophages stimulated with a range of macrophages in the presence or absence of AZ10606120 (10 µM) and quantified using AlamarBlue. **g** Light micrograph demonstrating the morphology of cells treated with 3 mM ATP in the presence or absence of AZ10606120 (10 µM). Experiments are representative of three independent experiments (*n* = 3). Error bars represent SD. Asterisks represent a significant difference (*p* < 0.05). Scale bars are 50 µm.

We have previously reported that CK could positively modulate P2X7^15^. P2X7 is expressed on J774 cells (Supplementary Fig. 1) and we confirmed that CK potentiates P2X7-induced Ca^2+^ responses. Pre-treatment with the selective P2X7 antagonist AZ10606120 abolished the ATP + CK mediated Ca^2+^ response (Fig. 1a, b). Furthermore, CK could enhance both the maximal ATP-induced Ca^2+^ response from J774 macrophages and caused a leftward-shift in the EC_50_, suggesting an increase in the sensitivity of cells to ATP (Fig. 1c). A characteristic feature of P2X7 activation is the formation of a large secondary pore, which allows impermeant molecules to traverse the membrane. Upon pore formation, YOPRO-1 can enter the cell, bind to DNA/RNA and fluoresce. CK could significantly potentiate ATP-induced YOPRO-1 dye uptake, enhancing both the maximal uptake of YOPRO-1 and reducing the concentration of ATP required to elicit this response (Fig. 1d, e).

P2X7 can contribute to inflammation via the regulation of cell death, therefore we investigated the effects of the positive modulator CK on enhancing macrophage cell death. It is well established that high concentrations ofATP in the mM range can induce cell death in numerous cell types expressing P2X7^1,2^. Following stimulation of J774 cells with a range of ATP concentrations 100 µM to 5 mM, only the 3 mM and 5 mM concentrations were capable of eliciting cell death (reducing cell viability to 29.4% and 38.3% of control, respectively; Fig. 1f, g). This effect could be reversed with AZ10606120 (10 µM) (Fig. 1f, g).

**Fig. 2:**
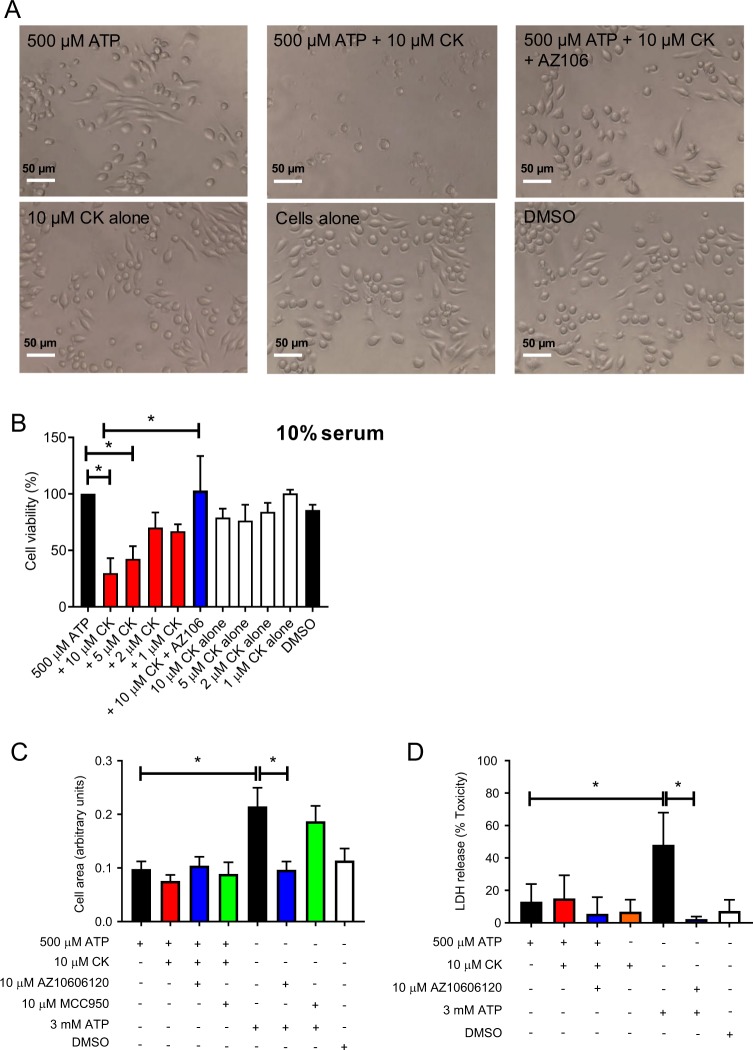
CK potentiated cell death is morphologically different to death induced by 3 mM ATP. **a** Light micrographs of J774 cells stimulated with 500 µM ATP alone or in the presence of various treatments (CK and AZ10606120 both 10 µM). **b** J774 cells were stimulated with decreasing concentrations of CK (10–1 µM) in combination with 500 µM ATP or alone. AZ10606120 was used at a concentration of 10 µM and cell death quantified by AlamarBlue. **c** J774 cells were stimulated with various treatments and the area of the cells after 3 h was quantified using Fiji. **d** LDH release following a 6 h stimulation with various treatments. Experiments are representative of three independent experiments (*n* = 3). Error bars represent SD. Asterisks represent a significant difference (*p* < 0.05). Scale bars are 50 µm.

To investigate the effect of CK on potentiation of P2X7-mediated cell death, we selected 500 µM ATP as it induces robust responses at P2X7 and is non-lethal (Fig. 1d). CK could not potentiate cell death to ATP concentrations lower than this (200 µM; Supplementary Fig. 2). We found that CK could enhance ATP-dependent cell death in a concentration-dependent manner, with 10 µM CK causing the most reduction in cell viability after 24 h (Fig. 2a, b). Moreover, CK had very little effect on cell viability on its own and therefore required the presence of ATP to elicit its cytotoxic effects (Fig. 2b). Notably, the cytotoxic effects of ATP in combination with 10 µM CK could be reversed by the addition of AZ10606120 (Fig. 2b).

Previous reports state that P2X7 activation in J774 macrophages by high ATP concentrations stimulates cell death via osmotic lysis, characterised by cell swelling and rupture^17^. In agreement, we show that cells exposed to 3 mM ATP swell to almost twice the size of cells exposed to 500 µM ATP and swelling was dependent on P2X7 but was not dependent on the NLRP3 inflammasome (Fig. 2c) as pre-treatment with the NLRP3 inhibitor, MCC950, had no effect (Fig. 2c). Cell swelling induced by 3 mM ATP was associated with a significant increase in the release of lactate dehydrogenase (LDH), which was not observed following stimulation of P2X7 by ATP + CK after 24 h (Fig. 2d).

### CK potentiates cell death in a caspase-dependent manner

**Fig. 3:**
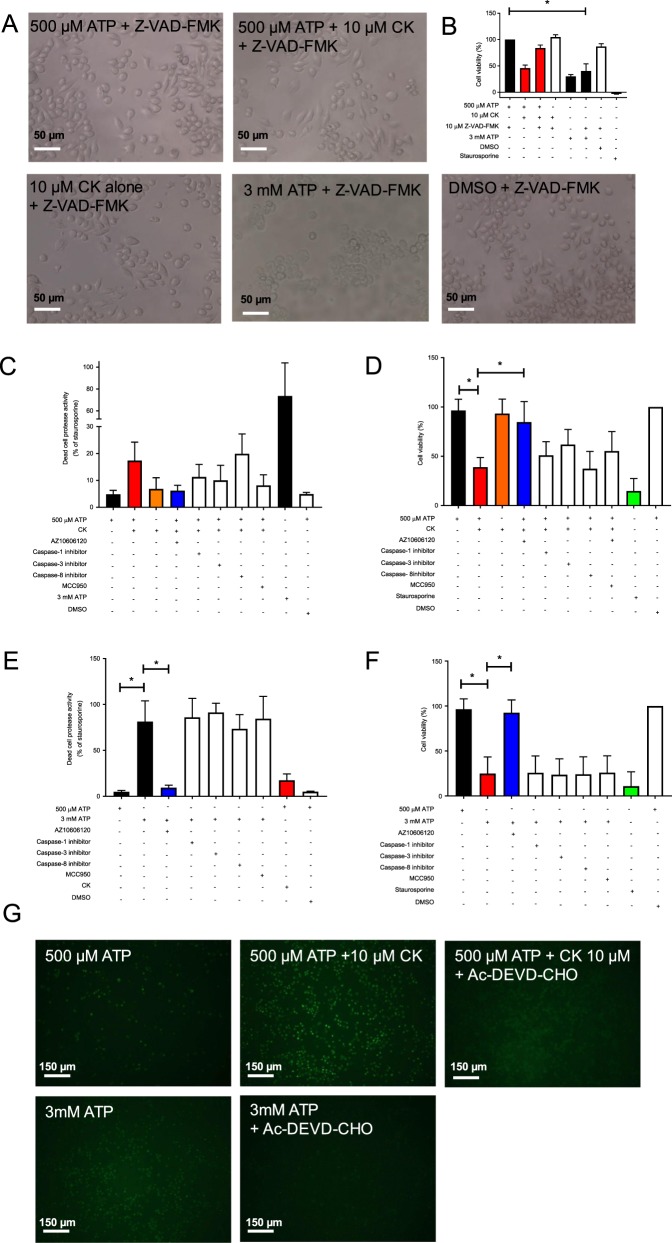
CK promotes cell death in a caspase-dependent manner but death induced by 3 mM ATP could not be blocked by caspase inhibitors. **a** Light micrographs depicting J774 cells pre-incubated with the pan-caspase inhibitor Z-VAD-FMK (10 µM) prior to stimulation with 500 µM ATP, 500 µM ATP + CK, CK alone, or 3 mM ATP. **b** Cell viability of J774 cells stimulated with the treatments outlined in **a**. **c** J774 cells were pre-incubated with inhibitors towards caspase-1, caspase-3, caspase-8, or MCC950 (all 10 µM) and challenged with ATP + CK (10 µM) for 6 h, prior to quantification of a protease released by dead cells. **d** J774 cells were pre-incubated with inhibitors towards caspase-1, caspase-3, caspase-8, or MCC950 (all 10 µM) and challenged with ATP + CK (10 µM) for 24 h, prior to quantification of cell death by Alamar Blue. **e** J774 cells were pre-incubated with inhibitors towards caspase-1, caspase-3, caspase-8, or MCC950 (all 10 µM) and challenged with 3 mM ATP for 6 h, prior to quantification of a protease released by dead cells. **f** J774 cells were pre-incubated with inhibitors towards caspase-1, caspase-3, caspase-8, or MCC950 (all 10 µM) and challenged with 3 mM ATP for 24 h, prior to quantification of cell death by Alamar Blue. **g** Fluorescent micrographs of J774 cells stimulated for 24 h with various treatments in the presence or absence of the caspase-3/7 inhibitor Ac-DEVD-CHO (10 µM). Caspase-3/7 activation was quantified using an EarlyTox™ Caspase-3/7 NucView 488 kit. Experiments are representative of three independent experiments (*n* = 3). Error bars represent SD. Asterisks represent a significant difference (*p* < 0.05).

We investigated whether cell death induced by 3 mM ATP or by ATP + CK was caspase dependent by pre-incubating with the pan-caspase inhibitor Z-VAD-FMK. Under these conditions, CK was unable to augment ATP-induced cell death (Fig. 3a, b), however the lytic cell death induced by 3 mM ATP was largely unaffected (Fig. 3a, b). We next proceeded to investigate the differences in P2X7-induced cell death by the two stimuli by using inhibitors of specific caspases. In order to quantify cell death at an earlier time-point (6 h) we utilised a multi-tox fluor cytotoxicity assay allowing us to measure live cells and dead cells simultaneously. After 6 h ATP + CK increased cell death by 3.6-fold (Fig. 3c), and this could be inhibited by AZ10606120. This ATP + CK-induced cell death was partly reversed by inhibitors for caspase-1, caspase-3, and MCC950, but not by inhibitors of caspase-8 (Fig. 3c). This trend was also observed over 24 h, although the effects of caspase inhibitors did not reach statistical significance (Fig. 3d). Conversely, death induced by 3 mM ATP was much more pronounced at the 6 h time-point with a 16.8-fold increase (Fig. 3e) and could not be reversed by any of the caspase inhibitors tested at either time-point (Fig. 3f). We measured activation of caspase-3/7 using a Nucview fluorescent substrate and found ATP + CK could stimulate caspase-3/7 activation and this was inhibited by the caspase-3 inhibitor Ac-DEVD-CHO (Fig. 3g).

### Kinetics of caspase-3/7 activation are regulated by P2X7

**Fig. 4:**
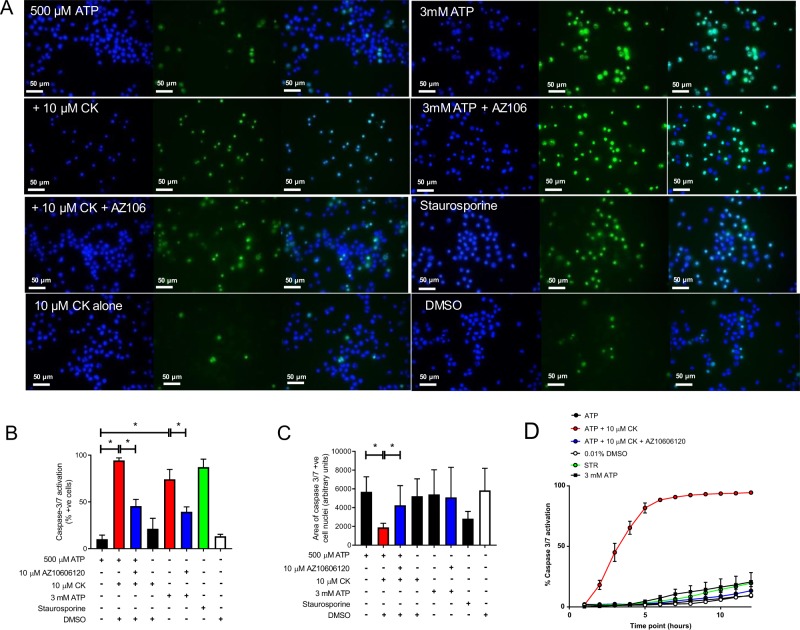
CK accelerates caspase-3/7 activation and promotes nuclear condensation in a P2X7-dependent manner. **a** J774 cells were stimulated for 24 h with 500 µM ATP alone, or in combination with CK or CK and AZ10606120 (both 10 µM), with 3 mM ATP or with CK alone. Staurosporine (5 µM) was used as a positive control. **b** The percentage of caspase-3/7 cells following each treatment after 24 h was quantified from fluorescent micrographs using Fiji. **c** The relative area of the caspase-3/7-positive cells from **a, b** was quantified using Fiji. **d** The kinetics of caspase-3/7 activation was reported following quantification of the caspase-3/7-positive cells every hour for 12 h using an ImageXpress. Experiments are representative of three independent experiments (*n* = 3). Error bars represent SD. Asterisks represent a significant difference (*p* < 0.05). Scale bars are 50 µm.

Numerous studies have implicated a role for P2X7 in the activation of pro-apoptotic caspases 8, 9, and 3^4^. Following stimulation of cells with ATP + CK, almost all the cells were caspase-3/7-positive after 24 h (93.2%) compared to ~10% for 500 µM ATP alone (Fig. 4a, b). Moreover, caspase-3/7 activation was inhibited by antagonism of P2X7 with a 43% decrease (Fig. 4a, b). Three millimolar ATP also induced substantial caspase-3/7 activation after 24 h in a P2X7-dependent manner, although the percentage of positive cells was lower (76.3%; Fig. 4a, b). CK alone (10 µM) induced a small increase in caspase-3/7 activation but this was not significant when compared to ATP or the vehicle control (Fig. 4a, b). We observed that cell nuclei were condensing following treatment with ATP + CK comparable to the effect of staurosporine, suggesting that cells might be undergoing classical apoptosis (Fig. 4a). Nuclei area was quantified using Fiji software (ImageJ) and ATP + CK treated cells showed a 3-fold reduction (Fig. 4c). In contrast, nuclear condensation was not observed following stimulation with 3 mM ATP (Fig. 4c).

Treatment of cells with ATP + CK not only maximised caspase-3/7 activation, it also significantly accelerated caspase-3/7 activation kinetics measured using a NucView-488 caspase-3/7 assay. Treatments with ATP alone, DMSO, 3 mM ATP, and staurosporine resulted in <15% caspase-3/7 activation in the first 8 h of stimulation (Fig. 4d). Conversely, ATP + CK stimulated caspase-3/7 activation within 30 min and led to almost 100% of cells becoming caspase-3/7-positive within 6 h (Fig. 4d). This effect was inhibited by AZ10606120 and CK itself did not elicit significant caspase-3/7 activation in this timeframe (Fig. 4d).

### CK can potentiate the generation of mitochondrial reactive oxygen species (mtROS) and loss of mitochondrial membrane potential

**Fig. 5:**
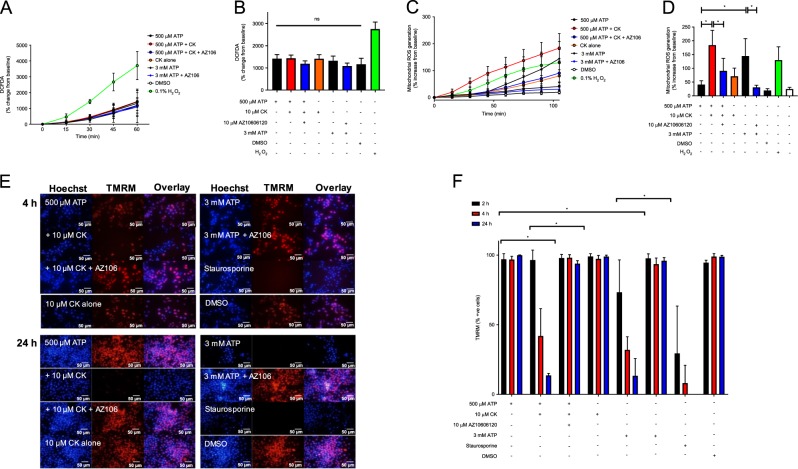
CK potentiates mtROS and loss of mitochondrial membrane potential but not cellular ROS generation. **a** J774 cells were pre-incubated with 5 µM DCFDA prior to the respective treatments and DCFDA fluorescence was measured every 15 min for 1 h. **b** The relative amount of DCFDA fluorescence as calculated by percentage change from the baseline fluorescence after 1 h, following the treatments in **a**. **c** J774 cells were pre-incubated with 5 µM mitoSOX prior to the respective treatments and mitoSOX fluorescence was measured every 15 min for 105 min. **d** The relative amount of mitoSOX fluorescence as calculated by percentage change from the baseline fluorescence after 105 min, following the treatments in **c**. Experiments are representative of three independent experiments (*n* = 3). Error bars represent SD. Asterisks represent a significant difference (*p* < 0.05). **e** J774 cells were stimulated for 4 h with 500 µM ATP alone, or in combination with CK or CK and AZ10606120 (both 10 µM), with 3 mM ATP ± AZ10606120, or with CK alone. Staurosporine (5 µM) was used as a positive control. Cells were incubated with TMRM (100 nM) for the final 30 min of the treatment time (4 h or 24 h) and fluorescent images were taken. **f** The percentage of cells positive for TMRM was identified by quantifying the number of cells positive for TMRM fluorescence in Fiji. Experiments are representative of three independent experiments (*n* = 3). Error bars represent SD. Asterisks represent a significant difference (*p* < 0.05). Scale bars are 50 µm.

Based on the rapid activation of caspase-3/7, cell shrinking and nuclei condensation, it would seem likely that the route of cell death induced by ATP + CK could be apoptotic. Apoptosis can be induced by a large number of extrinsic and intrinsic signals, including reactive nitrogen species, DNA damage, hypoxia, serum deprivation, and pathogenic challenge^18^. Moreover, the exposure to xenobiotics, such as pesticides, chemotherapeutic drugs, and phytochemicals, can trigger apoptosis, which are often mediated by reactive oxygen species (ROS)^19^. We first quantified the production of cellular ROS in response to P2X7 activation using the fluorescent indicator DCFDA. Here we observed that stimulating J774 macrophages with ATP + CK did not induce a significant increase in DCFDA fluorescence over 60 min compared to 3 mM ATP or CK alone (Fig. 5a, b) whereas the positive control (hydrogen peroxide) enhanced cellular DCFDA fluorescence. ATP + CK and 3 mM ATP-induced changes in DCFDA were not affected by AZ10606120 suggesting P2X7 does not contribute to cellular ROS production (Fig. 5a, b).

Mitochondrial ROS (mtROS) can stimulate an increase in mitochondrial membrane permeability, which allows the release of pro-apoptotic molecules such as cytochrome c and apoptosis-inducing factor into the cytosol. We therefore investigated changes in the production of mtROS and in mitochondrial membrane potential. mtROS was quantified over a 105 min stimulation period using mitoSOX and the combination of ATP + CK was capable of inducing an almost 4-fold increase in the production of mtROS compared to ATP alone (Fig. 5c, d). Unlike cellular ROS production, the potentiation of mtROS production by CK appeared to be predominantly regulated by P2X7, as inhibition of P2X7 reduced mtROS back to baseline values (Fig. 5c, d). Furthermore, akin to the caspase-3/7 observations, CK not only influenced the magnitude of mtROS generation, but also accelerated the production of mtROS (Fig. 5c). MtROS production was also induced by 3 mM ATP, although the rate of production was delayed compared to ATP + CK (Fig. 5c, d).

Changes in mitochondrial membrane potential were quantified using TMRM. Following stimulation with 500 µM ATP over 24 h, a high level of mitochondrial fluorescence was observed, indicating that TMRM was being retained in mitochondria and the membrane potential remained intact (Fig. 5e, f). Conversely, addition of ATP + CK, 3 mM ATP, or staurosporine resulted in a significant loss of TMRM fluorescence over time, indicating a loss of mitochondrial membrane potential (Fig. 5e, f). Mitochondrial membrane collapse by ATP + CK and 3 mM ATP, could be completely inhibited by AZ10606120 (Fig. 5e, f). Kinetically, 3 mM ATP could induce loss of mitochondrial membrane potential faster than ATP + CK, with 26.6% of cells losing membrane potential compared to 3.6% after 2 h stimulation, respectively. By 4 h the percentage of cells losing membrane potential increased to 58.1% and 68% for ATP + CK and 3 mM ATP, respectively. After 24 h, the loss of membrane potential between the two were comparable with 86.4% and 86.7% of cells losing membrane potential following stimulation with ATP + CK or 3 mM ATP, respectively (Fig. 5f).

### Cell death and loss of mitochondrial membrane potential induced by ATP + CK can be reduced by inhibiting Bax and scavenging mtROS

**Fig. 6:**
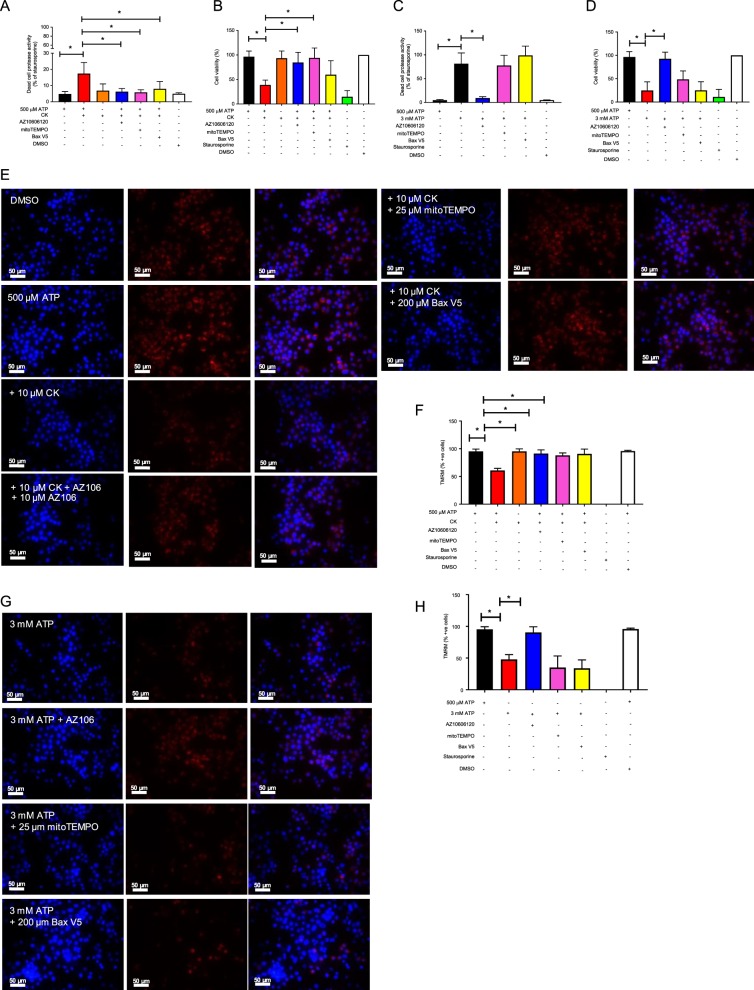
Scavenging mtROS or inhibiting Bax can prevent cell death and loss of mitochondrial membrane potential following stimulation with ATP + CK but not by 3 mM ATP. **a** J774 cells were pre-incubated with the mtROS scavenger mitoTEMPO (25 µM) or the Bax V5 inhibitor peptide (200 µM) and challenged with ATP + CK (10 µM) for 6 h, prior to quantification of a protease released by dead cells. **b** or a 24 h stimulation prior to quantification of cell death by AlamarBlue. **c** J774 cells were pre-incubated with the mtROS scavenger mitoTEMPO or the Bax V5 inhibitor peptide and challenged with 3 mM ATP for 6 h, prior to quantification of a protease released by dead cells. **d** or a 24 h stimulation prior to quantification of cell death by by AlamarBlue. **e** Fluorescent micrographs of J774 cells pre-incubated with the mtROS scavenger mitoTEMPO or the Bax V5 inhibitor peptide and challenged with ATP + CK for 4 h, prior to incubation with TMRM (100 nM) for the final 30 min of the treatment time to measure mitochondrial membrane potential. **f** The percentage of cells positive for TMRM after stimulation with ATP + CK was identified by quantifying the number of cells positive for TMRM fluorescence in Fiji. **g** Fluorescent micrographs of J774 cells pre-incubated with the mtROS scavenger mitoTEMPO or the Bax V5 inhibitor peptide and challenged with 3 mM ATP for 4 h, prior to incubation with TMRM (100 nM) for the final 30 min of the treatment time to measure mitochondrial membrane potential. **h** The percentage of cells positive for TMRM after stimulation with 3 mM ATP was identified by quantifying the number of cells positive for TMRM fluorescence in Fiji. Experiments are representative of three independent experiments (*n* = 3). Error bars represent SD. Asterisks represent a significant difference (*p* < 0.05). Scale bars are 50 µm.

We explored the mechanisms underlying mtROS generation and the loss of mitochondrial membrane potential correlated with differential cell death outcomes following P2X7 activation using mitoTEMPO as a scavenger of mtROS and a Bax inhibitory peptide to prevent Bax activation. After a 6 h incubation with ATP + CK, cell death could be completely inhibited by mitoTEMPO or the Bax inhibitory peptide (Fig. 6a). Over prolonged stimulations, mitoTEMPO could inhibit cell death to a similar level as AZ10606120, suggesting that mtROS was one of the early triggers of cell death under these conditions. However, the Bax inhibitor could not inhibit cell death as effectively after 24 h of stimulation (Fig. 6b). Conversely, neither mitoTEMPO nor the Bax inhibitory peptide could inhibit cell death induced by 3 mM ATP following either a 6 h or 24 h stimulation (Fig. 6c, d). These observations were also extended to mitochondrial membrane potential. Following 4 h of stimulation with ATP + CK, ~50% of cells had completely lost mitochondrial membrane potential (no TMRM fluorescence) and this was inhibited by mitoTEMPO and inhibition of Bax (Fig. 6e, f). Neither mitoTEMPO nor Bax inhibitory peptide could inhibit the loss of mitochondrial membrane potential elicited by 3 mM ATP (Fig. 6g, h).

### P2X7 stimulated increases in mitochondrial Ca^2+^ contribute to mtROS production and cell death

**Fig. 7:**
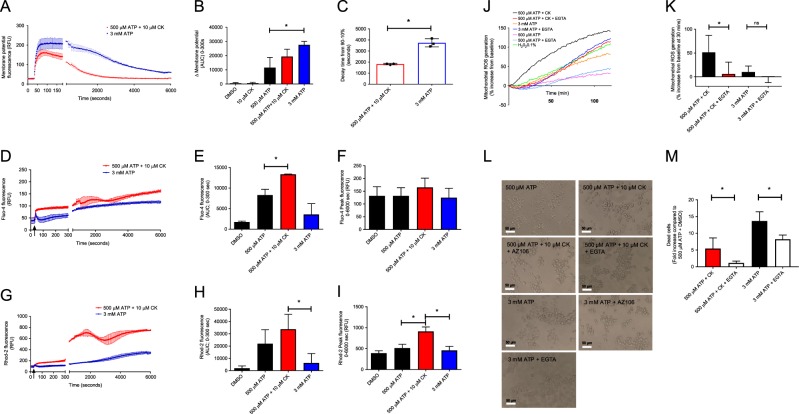
CK + ATP stimulates the accumulation of Ca^2+^ in the mitochondria which is critical for mtROS generation and apoptotic cell death. **a** Kinetic analysis quantifying the movement of charged ions across the cell membrane over two time periods totalling 6000 sec, following stimulation with ATP + CK or 3 mM ATP. **b** The initial movement of charged ions as quantified by area under the curve from 0–180 sec. **c** The time taken for the response to decrease from 90% of the maximal response to 10% of this depolarisation response. **d** The measurement of intracellular Ca^2+^ by quantification of Fluo-4 fluorescence over two time periods totalling 6000 s following stimulation with ATP + CK or 3 mM ATP. **e** The initial measurement of intracellular Ca^2+^ as quantified by area under the curve from 0–300 sec. **f** The peak Fluo-4 fluorescence reached over the 6000 sec time-period. **g** The measurement of mitochondrial Ca^2+^ by quantification of Rhod-2 fluorescence over two time periods totalling 6000 sec following stimulation with ATP + CK or 3 mM ATP. **h** The initial measurement of mitochondrial Ca^2+^ as quantified by area under the curve from 0–300 sec. **i** The peak Rhod-2 fluorescence reached over the 6000 s time-period. **j** mtROS production was measured using mitoSOX every 60 sec for 120 min following stimulation with ATP alone, ATP + CK, or 3 mM ATP in the presence or absence of 5 mM EGTA. **k** The % increase of mtROS generation from baseline level after 30 min in the presence or absence of EGTA following stimulation with ATP + CK, or **c** 3 mM ATP. **l** light micrographs depicting cell death in the presence or absence of EGTA and respective stimulations. **m** Cell death induced by ATP + CK or 3 mM ATP in the presence of Ca^2+^ compared to when Ca^2+^ is chelated by EGTA. All experiments are representative of three independent experiments (*n* = 3). Error bars represent SD. Asterisks represent a significant difference (*p* < 0.05). Scale bars are 50 µm.

Ca^2+^ plays an important role in regulating numerous biological processes, including the induction of mtROS. Therefore, we investigated the effect of Ca^2+^ influx on the generation of mtROS and mechanism of cell death. We first measured the total inward movement of charged ions (depolarisation) across the plasma membrane of J774 cells following stimulation of P2X7 with ATP + CK by utilising a cellular membrane potential kit. Activation of P2X7 by ATP + CK or 3 mM ATP evoked a large membrane depolarisation due to the inward flux of cations through open P2X7 channels (Fig. 7a). Measuring the first 180 sec of P2X7 activation revealed a larger depolarisation response amplitude to 3 mM ATP than to ATP + CK (Fig. 7b). Continued measurement of plasma membrane potential over 90 min revealed that the ATP + CK response was significantly shorter in duration than the 3 mM ATP-induced response with the mean time taken to fall from 90% to 10% response being 1820 sec (30.3 min) compared with 3740 sec (62.3 min) for 3 mM ATP (Fig. 7a, c). However, specifically measuring intracellular Ca^2+^ concentrations in cytosolic and mitochondrial compartments with Fluo-4 and Rhod-2 Ca^2+^ indicator dyes, respectively, identified that the cytosolic elevations in Ca^2+^ and mitochondrial uptake were significantly greater following the application of ATP + CK compared to 3 mM ATP (Fig. 7d–i). Continued measurement of Ca^2+^ elevations in cytosol and mitochondria over 90 min revealed larger and more sustained elevations in Ca^2+^ with ATP + CK (Fig. 7c–i).

Enhanced mitochondrial Ca^2+^ can be a trigger to produce mtROS, and mtROS appeared to be the trigger for cell death induced by positive modulation of P2X7. Chelation of extracellular Ca^2+^ using EGTA resulted in both a decreased and delayed induction of mtROS by ATP + CK but did not affect the slower production of mtROS following stimulation with 3 mM ATP (Fig. 7j). Focusing on data obtained at the 30 min time-point showed that removing Ca^2+^ could significantly abrogate the quick onset of mtROS generation by ATP + CK but not 3 mM ATP (Fig. 7k). Chelating extracellular Ca^2+^ could significantly inhibit the cell death induced by 3 mM ATP, but inhibited the cell death induced by ATP + CK to a greater extent after 6 h suggesting that Ca^2+^ plays a critical role in CK-potentiated apoptosis (Fig. 7m).

## Discussion

**Fig. 8:**
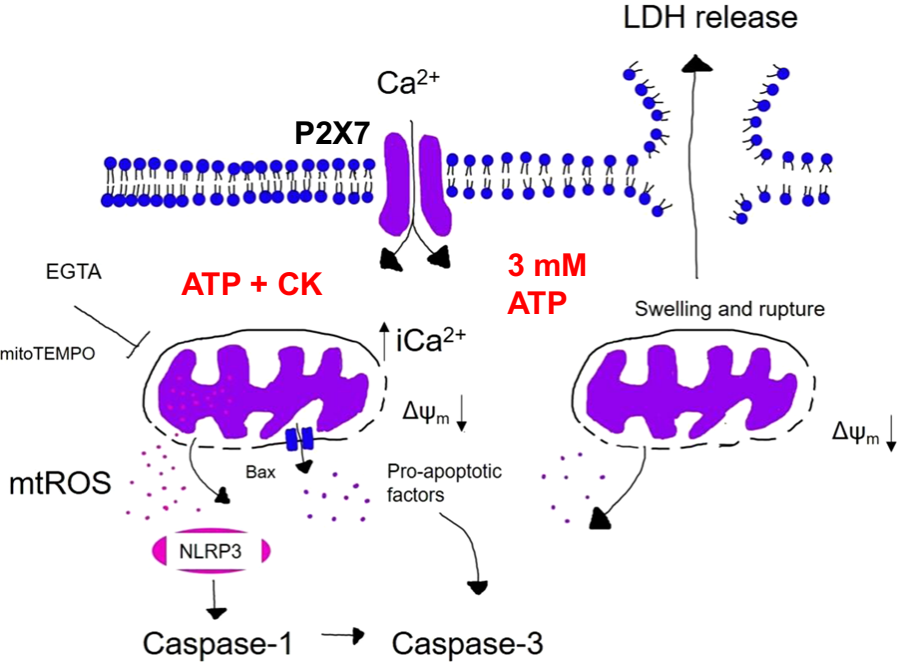
Summary of P2X7-dependent signals eliciting different cell death responses. Upon stimulation with 3 mM ATP, J774 cells die in a caspase-independent manner. Here, the movement of charged ions into the cell disrupts the osmotic equilibrium and cells begin to swell. This leads to loss of mitochondrial membrane potential and membrane rupture, releasing LDH to the extracellular milieu. Cells that have not ruptured then become caspase-3/7-positive. However, stimulation with ATP + CK results in an increase in mitochondrial Ca^2+^ that stimulates the production of mtROS. This results in cell death that is both dependent upon Bax and caspase activation. This cell death could also be inhibited by scavenging mtROS or chelating Ca^2+^ using mitoTEMPO or EGTA, respectively. In this instance, it could be possible that mtROS might interact with the NLRP3 inflammasome and activate caspase-1, which in turn might cleave caspase-3 and explain the significantly accelerated caspase-3/7 activation observed following stimulation with ATP + CK.

Our study focused on understanding the mechanism of cell death induced following positive allosteric modulation of the ATP-gated ion channel, P2X7. As a result, we demonstrated several findings. Notably, high millimolar ATP concentrations stimulated an unregulated form of cell death, which could not be stopped by inhibiting caspases, blocking Bax, or by scavenging mtROS, and was characterised by early cell swelling and cell lysis. Conversely, the combination of ATP and CK induced a significant increase in mitochondrial Ca^2+^, which was accompanied by accelerated mtROS production, accelerated caspase-3/7 activation, and cell death that can be partly inhibited by caspase inhibitors, inhibition of Bax, removal of extracellular Ca^2+^, or mtROS scavenging (Fig. 8).

Maximal ATP (3 mM) could elicit a large movement of charged ions (depolarisation) but CK-induced potentiation of P2X7 appeared to selectively increase Ca^2+^ influx and intracellular Ca^2+^ concentrations, notably enhancing mitochondrial Ca^2+^. We have previously reported in detail that CK can act as a positive allosteric modulator of P2X7 receptors^15,16^. Utilising a molecular modelling and mutagenesis approach we recently identified an allosteric pocket within the central vestibule of P2X7. However, it is still currently poorly understood how binding to this novel binding pocket enhances ion channel activity, macropore formation, or how CK modulates ion permeability^15,16^.

It is well established that P2X7 can participate in the regulation of cell death, with high concentrations of ATP inducing necrotic, pyroptotic, or apoptotic cell death depending on the stimulatory factors present, the incubation time, and the cell type^3,11,20,21^. Previous reports state that P2X7 activation stimulates cell death in J774 macrophages via colloido-osmotic lysis, characterised by cell swelling and rupture^17^. Concordant with these observations, J774 cells treated with 3 mM ATP swelled to almost twice the size and appeared to rupture, based on the detection of LDH in the cell supernatant. Moreover, this lytic cell death could not be inhibited by caspase inhibitors, mtROS scavenging, or Bax inhibition. However, chelation of extracellular Ca^2+^ could prevent some of the cell death induced by 3 mM ATP, which suggests the large movement of ions and divergence from the osmotic equilibrium are likely key contributors to this form of cell death.

Conversely, the fast increase in mitochondrial Ca^2+^ and subsequent production of mtROS seem to be critical triggers in the induction of cell death following stimulation with ATP + CK. The accelerated kinetics of mtROS are correlated with caspase-3/7 activation and likely occurs because of the loss of mitochondrial membrane integrity induced by mtROS or via release of pro-apoptotic factors through Bax channels. Moreover, this form of cell death was caspase-dependent, and the pan-caspase inhibitor could limit cell death. Inhibition of individual caspases had less of an effect but the NLRP3 inflammasome, caspase-1, and caspase-3 were implicated. MtROS production is linked to activation of the NLRP3 inflammasome^22^, and caspase-1 has recently been identified to activate caspase-3^23^. Additional activation of caspase-3 by caspase-1 could explain why activation of caspase-3/7 was so significantly accelerated, but this is something that would need to be explored further.

There is growing evidence that ROS and mitochondria play an important role in stimulating apoptosis, whilst mitochondria are also both a target and source of ROS^19^. P2X7 activation has been implicated in the generation of ROS in macrophages and microglia, which could be inhibited by P2X7 antagonists including oxidized ATP, Brilliant Blue G, and AZ10606120^5,24,25^. In contrast to cellular ROS, P2X7 activation and Ca^2+^ were critical in the generation of mtROS following stimulation with ATP and CK. Notably, ATP and CK could stimulate an instant spike in mtROS production and upon removal of Ca^2+^ or inhibition of P2X7, this initial spike was lost, and cells survived.

ROS initiate the intrinsic pathway of mitochondria and promote the activation of pro-apoptotic proteins^26–28^. An increase in the production of mtROS, particularly by hydrogen peroxide, has previously been directly linked with collapse of mitochondrial membrane potential^29–31^. P2X7 has been implicated in stimulating mitochondrial dysfunction and membrane collapse in response to large increases in intracellular Ca^2+^ accumulation. The subsequent loss of mitochondrial membrane potential and increase in mitochondrial membrane permeability has been implicated in the release of numerous pro-apoptotic factors such as apoptosis-inducing factor and cytochrome c, which contributes to formation of the apoptosome complex, activation of caspase-3, and ultimately apoptosis^32–34^. Therefore, by reducing the amount of Ca^2+^ available to enter the cell, we have limited the ability of ATP and CK to promote the steps necessary to evoke early mtROS production, thus removing the initial trigger and the ability of CK to promote cell death.

To conclude, CK potentiation of P2X7-dependent responses promotes the induction of the intrinsic pathway of apoptosis via an increase in mitochondrial Ca^2+^, accelerated production of mtROS, loss of mitochondrial membrane potential, and accelerated caspase-3/7 activation while activation with high ATP concentrations induces an early caspase-independent lytic cell death pathway. This provides evidence that allosteric modulation of P2X7 can be exploited to calibrate cell death outcomes. Positive allosteric modulation of P2X7 receptors opens various avenues of exploration; such as whether it can enhance the killing of intracellular pathogens, augment the removal of P2X7 expressing cancer cells, or contribute to the resolution of inflammation.

## Supplementary information

Supplementary Figure 1

Supplementary Figure 2

Supplementary figure legends

## Data Availability

Data are available upon reasonable request to the corresponding author.
