# Supplementary figures and images for: Positive allosteric modulation of P2X7 promotes apoptotic cell death over lytic cell death responses in macrophages
Source: Cell Death Dis. 2019 Nov 25;10(12):882. doi: 10.1038/s41419-019-2110-3 (PMC6877589; doi:10.1038/s41419-019-2110-3)

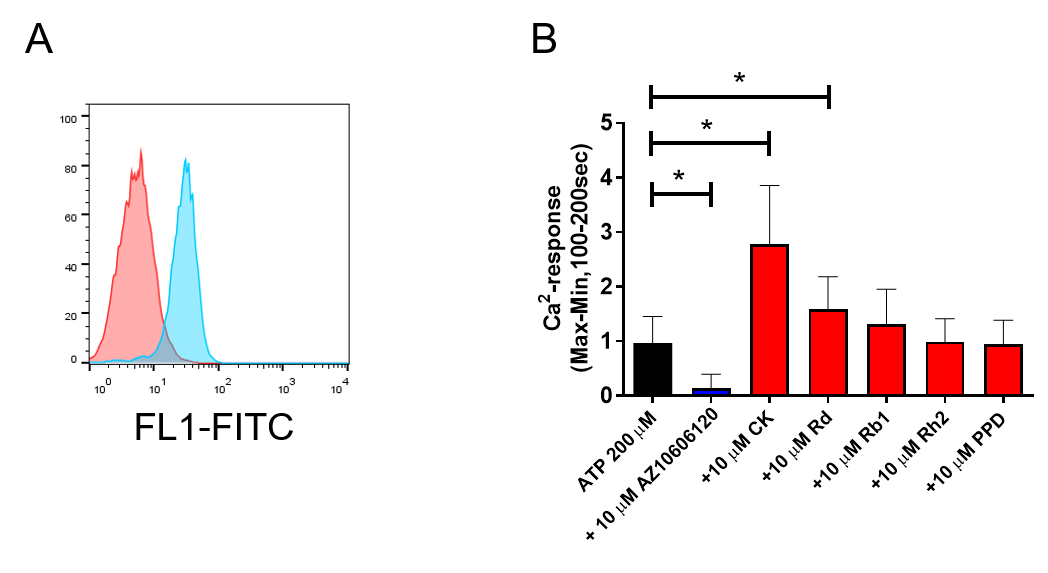

Supplement: Supplementary file 1 — Supplementary Figure 1 [file 41419_2019_2110_MOESM1_ESM.tif]

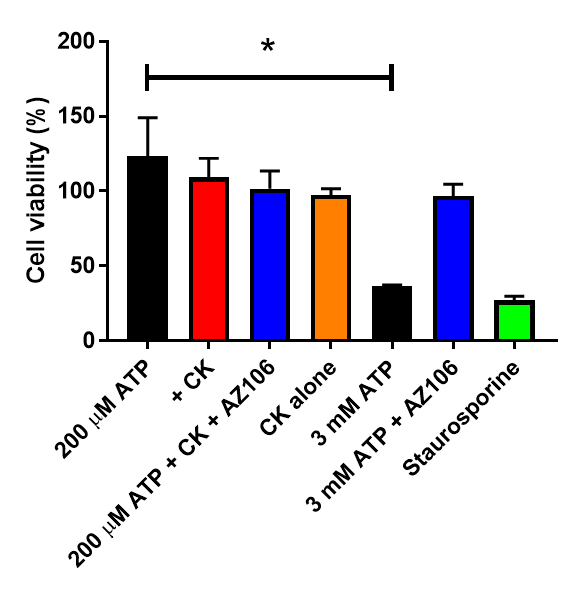

Supplement: Supplementary file 2 — Supplementary Figure 2 [file 41419_2019_2110_MOESM2_ESM.tif]
