# Supplementary material for: Positive allosteric modulation of P2X7 promotes apoptotic cell death over lytic cell death responses in macrophages
Source: Cell Death Dis. 2019 Nov 25;10(12):882. doi: 10.1038/s41419-019-2110-3 (PMC6877589; doi:10.1038/s41419-019-2110-3)
Supplement: Supplementary file 3 — Supplementary figure legends [file 41419_2019_2110_MOESM3_ESM.docx]

**Supplementary Figure 1:** **CK can potentiate the P2X7 responses on J774 macrophages. a** Flow cytometry histogram depicting the fluorescence (FL-1-FITC) of J774 cells stained with an anti-mouse P2X7 antibody (blue peak) compared to unstained cells (red). **b** Sustained intracellular Ca^2+^ responses obtained from J774 cells stimulated with 200 µM ATP in the presence or absence of 10 µM AZ10606120 or the ginsenosides CK, Rd, Rb1, Rh2, PPD. Experiments are representative of three independent experiments (*n=3*). Error bars represent SD. Asterisks represent a significant difference (p<0.05).

**Supplementary Figure 2: Potentiation of 200 µM ATP by CK is not sufficient to induce cell death of J774 cells.** Viability of J774 cells stimulated with 200 µM ATP in the presence or absence of 10 µM CK, CK alone, or CK and 10 µM AZ10606120. Or following stimulation with 3 mM ATP in the presence or absence of AZ10606120. Experiments are representative of three independent experiments (*n=3*). Error bars represent SD. Asterisks represent a significant difference (p<0.05).
